# KLF4 regulates TERT expression in alveolar epithelial cells in pulmonary fibrosis

**DOI:** 10.1038/s41419-022-04886-7

**Published:** 2022-05-04

**Authors:** Hui Wang, Hongyang Xu, Wenting Lyu, Qinghua Xu, Shiwen Fan, Haoran Chen, Dongjin Wang, Jingyu Chen, Jinghong Dai

**Affiliations:** 1 Department of Pulmonary and Critical Care Medicine, The Affiliated Drum Tower Hospital of Nanjing University Medical School, Nanjing, Jiangsu China; 2 Department of Critical Care Medicine, The Affiliated Wuxi People’s Hospital of Nanjing Medical University, Wuxi, Jiangsu China; 3 Department of Cardiothoracic Surgery, The Affiliated Drum Tower Hospital of Nanjing University Medical School, Nanjing, Jiangsu China; 4 Lung Transplant Center, The Affiliated Wuxi People’s Hospital of Nanjing Medical University, Wuxi, Jiangsu China

**Keywords:** Respiratory tract diseases, Ageing, Senescence

## Abstract

Idiopathic pulmonary fibrosis (IPF) was considered as a telomere-mediated disease. *TERT* and *TERC* correlated with telomere length. Although telomerase gene mutations were associated with IPF, majority patients did not carry mutations. The mechanism by which telomerase expression was regulated in IPF are still unclear. In this study, we aimed to delineate the mechanisms that how TERT protein expression were regulated in alveolar epithelial cells (AECs) in pulmonary fibrosis. Here, we found that P16, P21 and fibrosis markers (αSMA and Collagen-I) were prominently increased in lung tissues of IPF patients and bleomycin-induced mouse models, while the expression of KLF4 and TERT were decreased in AECs. In *vivo* experiments, AAV-6 vectors mediated KLF4 over-expression with specific SP-C promoter was constructed. Over-expression of KLF4 in AECs could protect TERT expression and suppress the development of pulmonary fibrosis in bleomycin-induced mouse models. In the mechanism exploration of TERT regulation, KLF4 and TERT were both down-regulated in bleomycin-induced senescent MLE-12 and BEAS-2B cells. Compared with control group, small-interfering RNA targeting KLF4 significantly reduced the TERT expression and telomerase activity, while overexpression of KLF4 can increased the expression of TERT and telomerase activity in senescent AECs. Furthermore, ChIP showed that KLF4 protein could bind to the TERT promoter region in MLE-12 cells, suggesting that KLF4 could implicate in pathogenesis of lung fibrosis through regulating TERT transcription in AECs. Taken together, this study identified that KLF4 might be a promising potential target for further understanding the mechanism and developing novel strategy for the treatment of lung fibrosis in IPF.

## Introduction

Idiopathic pulmonary fibrosis (IPF) is a lung fibrosis disease which is characterized by progressive dyspnea and impaired pulmonary function with an average survival about 3 years from diagnosis [1–3]. It was considered as an age-related disease since it occurs primarily in crowd over 60 years old, indicating senescence played a crucial role in the development of IPF [4–6]. IPF was one of the most common manifestations of telomere-mediated diseases [7]. Telomeres were defined as the specialized DNA–proteins complexes locating at the ends of chromosomes and could prevent chromosomes from degradation and genomic instability [7, 8]. Telomerase reverse transcriptase (TERT) could lengthens telomeres and was associated with senescence [9, 10]. Our previous study showed that IPF patients with TERT mutations usually accompanied with shorter telomeres [11]. However, only less than 10% of IPF patients carry telomerase gene mutations, while the vast majority of IPF patients, especially sporadic patients who deny family history, have decreased telomerase activity and shortened telomere length, suggesting that the mechanism by which telomerase expression and telomere length was regulated in IPF was still unclear [11–14].

Krüpple-like-factors were known as protein transcription factors and regulate diverse cell processes [15–17]. KLF4, one of them, has been shown to be one of four transcriptional factors (OCT3/4, SOX2, KLF4, and c‐MYC) essential for reprogramming different kinds of differentiated cells into induced pluripotent stem cells and was reported mainly expressed in the gastrointestinal and epithelial cells [18]. Its function depends on the interacting protein and binding site environment. It was considered as a transcriptional activator or repressor and played an important role in cell stemness [19]. Recent studies have shown that KLF4 was decreased in lung cancer and pulmonary arterial hypertension [20]. KLF4 was reported that could inhibited epithelial-mesenchymal transition (EMT) in renal epithelial cells, hepatocellular carcinoma cells, and mouse lung epithelial LA-4 cells [21]. Studies showed that knockdown of KLF4 promoted the activation of pathways above mentioned and TGF-β1-induced EMT, indicating that KLF4 plays an important role in bleomycin-induced lung fibrosis through suppressing TGF β1-induced EMT [22]. However, KLF4’s role in alveolar epithelial cells (AEC) in lung fibrosis is still not clear. Hoffmeyer et al. reported that KLF4 plays an important role in the regulation of telomerase gene expression [23]. Depletion of KLF4 in hESCs decreased the TERT protein expression [19]. KLF4 is required for maintaining hTERT expression in hESCs by directly activating its transcription. Its function in human AECs during pulmonary fibrosis progression remains unclear. Thus, we hypothesized that KLF4 may modulate TERT protein expression and telomere length and activity in AECs.

In this study, we demonstrated that the expression of KLF4 was decreased in AECs of human IPF and mouse models of bleomycin-induced pulmonary fibrosis. Overexpressing of KLF4 inhibited bleomycin-induced pulmonary fibrosis could protect TERT expression and telomere in AECs. These results provide further evidence that KLF4 represents a potential therapeutic target in IPF.

## Results

### Decreased TERT and KLF4 expression in AECs in IPF lung tissues

**Fig. 1:**
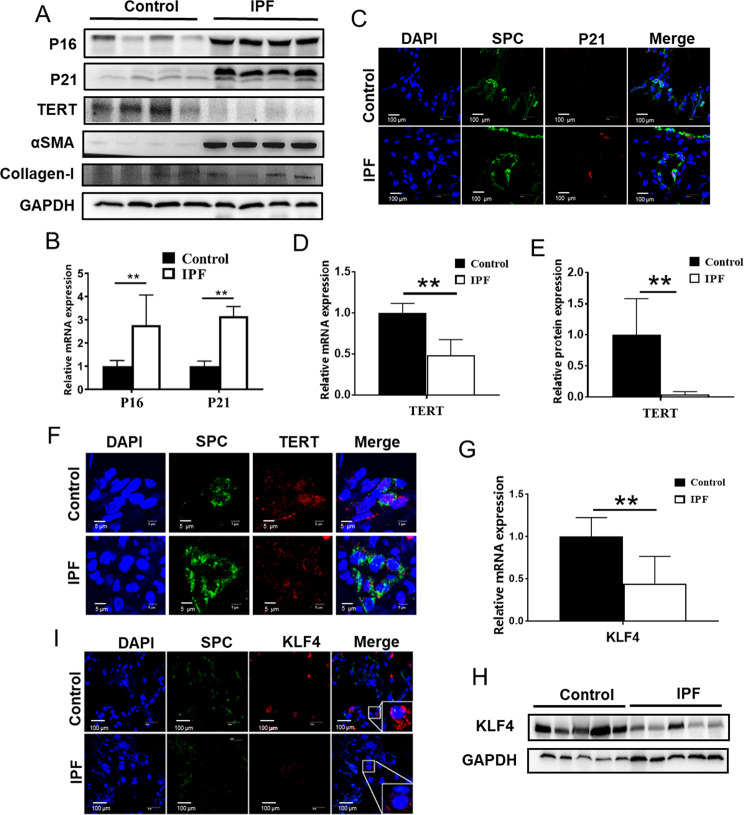
KLF4 and TERT expression was downregulated in AECs in human IPF lung tissues. **A** Western blotting was performed to evaluate the expression of P16, P21, TERT, αSMA and Collagen-I in IPF lung tissue and control lung tissues. Four randomly selected sample from each group were shown. **B**, **D** qPCR were performed to test P16, P21 and TERT in human IPF lung tissues and control group. **C** Double-labeled immunofluorescent staining were performed to examine the expression of SP-C (green) and P21 (red) in AECs. **E** Quantification of the protein level of TERT. **F** Double-labeled immunofluorescent staining were performed to examine the expression of SP-C (green) and TERT (red) in AECs. **G**, **H** qPCR and western blotting were performed to test KLF4 expression level in human IPF lung tissues and control group. **I** Immunofluorescent staining were performed to examine the expression of SP-C (green) and KLF4 (red) in AECs. *P < 0.05, **P < 0.01, ***P < 0.001 by *t*-test.

Previous studies showed that AECs in IPF patients underwent senescence and aging markers, such as P16, P21, were highly expressed in these cells [24–26]. To determine senescence markers and TERT expression in IPF, lung tissue sections from 12 IPF patients and 12 lung cancer adjacent normal tissues were acquired to undergo Western Blot and qPCR. IPF lung tissues were derived from IPF patients who received lung transplant. The demographic data was listed in Supplementary Table S1. The expression of P16, P21, αSMA and Collagen-I in IPF lung tissue was prominently increased and TERT expression was decreased (Fig. 1A, B). With double-label immunofluorescent staining method, to assess the presence of P21 in AECs, expression levels of SP-C (a specific marker of AEC) and P21 were analyzed in lung tissues from IPF and lung cancer adjacent normal group. Double-stained cells were detected in IPF (Fig. 1C). Very few double-positive cells were observed in the normal group (Fig. 1C). In contrast, the expression of TERT was decreased in IPF lung tissues, compared to the normal group (Fig. 1A, D, E). In normal lung tissues, TERT was mainly expressed in the nucleus in AECs (Fig. 1F). Consistent with the TERT expression, we also found the expression KLF4 was significantly decreased in IPF lung (Fig. 1G, H). Double-label immunofluorescent staining method showed that KLF4 was also decreased in AECs in IPF (Fig. 1I). It showed that the decreased levels of KLF4 might play important roles in the senescence of AECs in IPF.

### Decreased TERT and KLF4 expression in bleomycin-induced mouse lung tissues

**Fig. 2:**
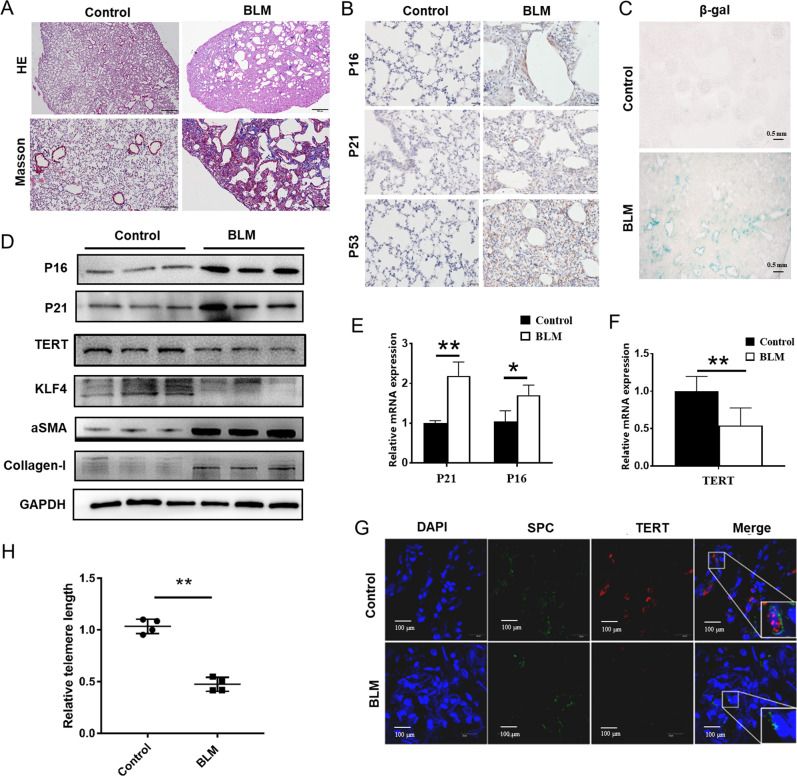
Decreased TERT and KLF4 expression in bleomycin-induced mouse lung tissues. **A** Representative microphotographs of H&E and Masson stainings of mouse models of bleomycin-induced pulmonary fibrosis group and control saline group. **B** Representative microphotographs of immunohistochemistry staining of P16, P21 and P53 in bleomycin-induced pulmonary fibrosis mouse model and control group. **C** β-gal staining was performed to mark senescent cells in lung tissue in bleomycin-induced mouse model and control group. **D** Western blotting was performed to evaluate the expression of P16, P21, KLF4, TERT, αSMA and Collagen-I expression in bleomycin-induced pulmonary fibrosis mouse and control group. **E**, **F** qPCR were performed to test P16, P21 and TERT in bleomycin-induced pulmonary fibrosis mouse and control group. **G** Double-labeled immunofluorescent staining were performed to examine the expression of SP-C (green) and TERT (red) in AECs between bleomycin-induced pulmonary fibrosis and control saline group. **H** Relative telomere lengths in lung tissues were tested in bleomycin group and control group. *P < 0.05, **P < 0.01, ***P < 0.001 by *t*-test.

Then we tested KLF4 expression and senescence markers in pulmonary fibrosis old mouse (6 months old) models induced by bleomycin tracheal instillation. The mice were randomly divided into two groups (control and bleomycin group) by random number table. As anticipated, the lung sections from bleomycin-treated mice for 14 days displayed marked alveolar damage, structural destruction, and accumulation of fibrotic collagens in the alveolar parenchyma was shown in H&E and Masson stains (Fig. 2A). The immunohistochemistry showed that the expressions of senescence markers P16, P21 and P53 significantly increased in bleomycin-induced mouse lung tissues compared with the control group (Fig. 2B). We also found that increased positive staining of SA-β-gal was located in the epithelial area in fibrosis lung tissues, which was hardly seen in normal lung tissues (Fig. 2C). Consistent with the results in human lung tissues, the expression of P16, P21 were increased and KLF4, TERT were decreased in bleomycin-induced pulmonary fibrosis. qPCR and western blotting were performed and confirmed that expression of TERT was down-regulated and P16, P21 together with fibrotic markers (αSMA and Collagen-I) were increased in bleomycin-induced pulmonary fibrosis tissues at both mRNA and protein levels (Fig. 2D–F). Double-label immunofluorescent staining method showed that TERT was decreased in AECs in bleomycin-induced pulmonary fibrosis (Fig. 2G). Moreover, telomere length was also tested and bleomycin-induced pulmonary fibrosis lung tissues had shorten telomere length. The results showed that AECs in bleomycin-induced pulmonary fibrosis model mice underwent senescent, and the expression of KLF4 and TERT was significantly decreased.

### Overexpression of KLF4 attenuated senescence in bleomycin-induced pulmonary fibrosis model

**Fig. 3:**
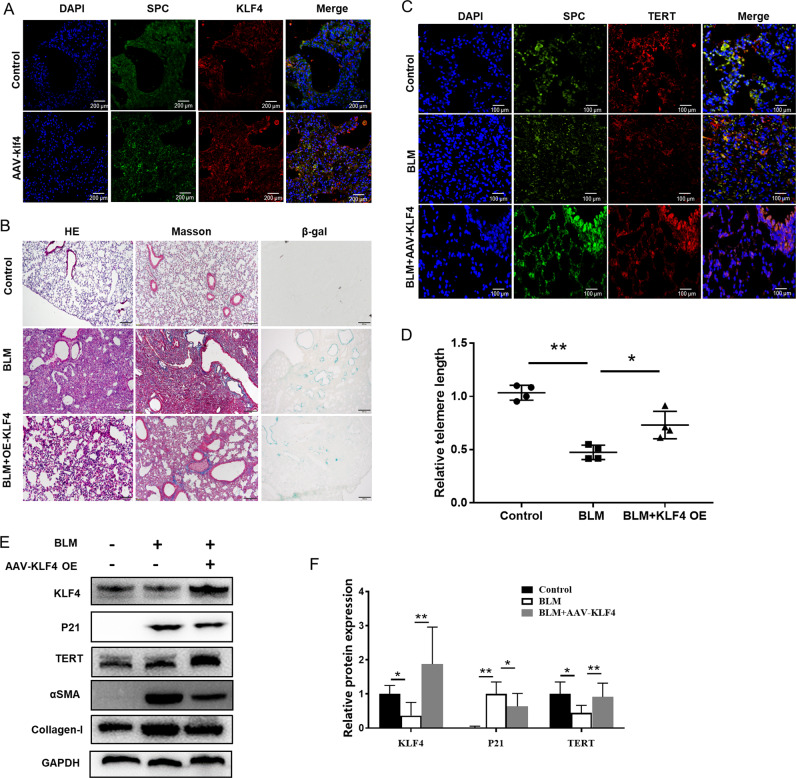
Overexpression of KLF4 attenuated senescence and fibrosis in bleomycin-induced pulmonary fibrosis model. Adeno-associated viral (AAV)-6 vectors mediated KLF4 over-expression with SP-C promoter was constructed and it can increase the expression of KLF4 in AECs. **A** Overexpression of KLF4 in the AAV-6 injected mice were confirmed with double-labeled immunofluorescent staining. Mice were intratracheally intubated and injected with bleomycin to induce pulmonary fibrosis. Saline was used as a control, in overexpression of KLF4 mice group and control group. **B** H&E and Masson staining were used to detect collagen depositions. β-gal staining was performed to mark senescent cells in lung tissue. **C** Double-labeled immunofluorescent staining were performed to examine the expression of SP-C (green) and TERT (red) in AECs in three groups. **D** Relative telomere lengths in lung tissues were tested. **E** Western blotting was performed to evaluate the expression of P21, KLF4, TERT, αSMA and Collagen-I expression in KLF4 overexpressing group and control group. **F** Quantification of the protein level of P21, KLF4 and TERT in lung tissues. *P < 0.05, **P < 0.01, ***P < 0.001 by *t*-test.

To investigate the effect of KLF4 on pulmonary fibrosis in vivo, the adeno-associated viral (AAV)−6 vectors mediated KLF4 over-expression with SP-C promoter was constructed, which can specifically target on AECs and improve the expression level of KLF4 in AECs. The AAV-6 was injected into mouse before bleomycin infected. The expression of KLF4 in AECs was observed to be significantly up-regulated in AECs in KLF4-overexpressing group (Fig. 3A). The mice was administrated with intratracheal instillation of bleomycin in both KLF4-overexpressing mice and wild-type group. Severe lung fibrosis was present in bleomycin-administered wild-type mice (Fig. 3B). H&E, Masson and β-gal staining indicated that the severity of lung fibrosis and collagen fiber accumulation were decreased in KLF4-overexpressing group (Fig. 3B). The expression of TERT in the AECs of KLF4 overexpressing mice was higher than that of the control group in immunofluorescence (Fig. 3C). Besides, telomere length in overexpression group was longer than the control group, indicating that KLF4 could protect telomere length in pulmonary fibrosis. Additionally, the expression of TERT was down-regulated and P21 was up-regulated in bleomycin-induced pulmonary fibrosis model, compared with the control group (Fig. 3E). In KLF4-overexpressing group, the down-regulation of TERT was attenuated and up-regulation of P21 was inhibited compared with bleomycin group, while fibrotic markers (αSMA and Collagen-I) were reduced (Fig. 3E, F). The results suggest that KLF4 has a protective effect on pulmonary fibrosis. These results demonstrated that activation of KLF4 in epithelial cells could suppress the development of pulmonary fibrosis via impairing the activation of pulmonary fibroblasts.

### RNA Interference of KLF4 enhanced senescence in AECs

To further confirm the role of KLF4 in AECs, we examined the effect of KLF4 on TERT and senescence both in MLE-12 cells and BEAS-2B cells.

**Fig. 4:**
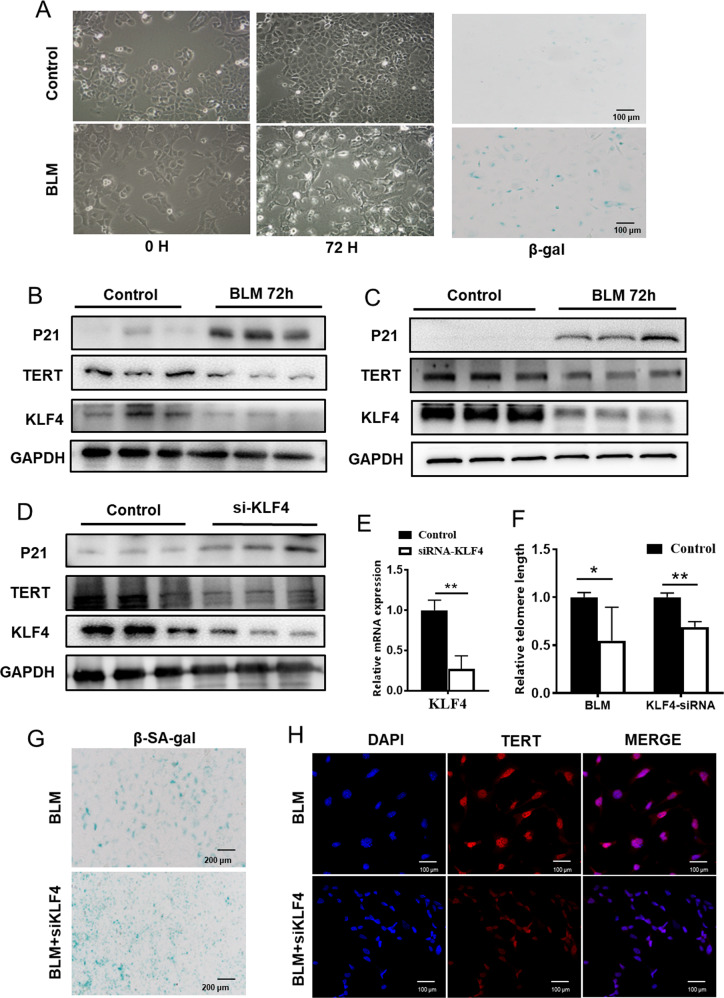
Knockdown of KLF4 induced senescence in AECs. MLE-12 cells were treated with 10 μg/mL bleomycin in culture medium for 12, 24, 48 and 72 h. **A** At 72 h, MLE-12 cells became enlarged, vacuolated, and sometimes appear with multiple or enlarged nuclei and β-gal stain was preformed to mark senescent cells. **B**, **C** Western blotting was performed to evaluate the expression of P21, KLF4 and TERT expression in bleomycin group and control group (**B** BEAS-2B cells; **C** MLE-12 cells). **D** MLE-12 cells were transfected with KLF4 siRNAs or control siRNA (100 nM). Western blotting was performed to evaluate the expression of P21, KLF4 and TERT expression in siRNA group and control group. **E** KLF4 expression level was assessed with qRT-PCR. **F** Telomere lengths were tested after constant stimulation of bleomycin in siRNA and control group. **G** β-gal stain was preformed to mark senescent cells after 72 h stimulation of bleomycin in siRNA group and control group. **H** Double-labeled immunofluorescent staining were performed to examine the expression of SP-C (green) and TERT (red) in these two groups. *P < 0.05, **P < 0.01, ***P < 0.001 by *t*-test.

MLE-12 cells and BEAS-2B cells/ were treated with 10 μg/mL bleomycin in culture medium for 12, 24, 48 and 72 h. After 72 hours, MLE-12 cells became enlarged, vacuolated, and sometimes appear with multiple or enlarged nuclei and SA-β-gal staining and β-galactosidase activity measurement showed that bleomycin increased the number of senescent cells and enhance the activity of β-galactosidase (Fig. 4A). In addition, the expression of P21 was increased and TERT was decreased in bleomycin-stimulated BEAS-2B cells (Fig. 4B) and MLE-12 cells (Fig. 4C). The results indicated that the senescent epithelial cell model in vitro was successfully constructed.

To examine whether endogenous KLF4 plays a role for TERT regulation in AEC, we transfected AECs with KLF4 siRNAs or control siRNA. KLF4 siRNA was shown to efficiently decrease KLF4 mRNA level (Fig. 4D, E). We also found that the expression of KLF4 was down-regulated after the injected of bleomycin (Fig. 4B, C). Besides, we found that interference of KLF4 significantly reduced the TERT expression level and telomerase length of AECs compared with the scramble group (Fig. 4D, F). TERT expression in the nucleus was decreased, and there were more senescent cells in RNA interference group in β-gal staining (Fig. 4G, H). These results suggest that the expression of KLF4 can affect TERT expression, and play a crucial role in the regulation of TERT.

### Overexpression of KLF4 Attenuated senescence in AECs and KLF4 regulate TERT

**Fig. 5:**
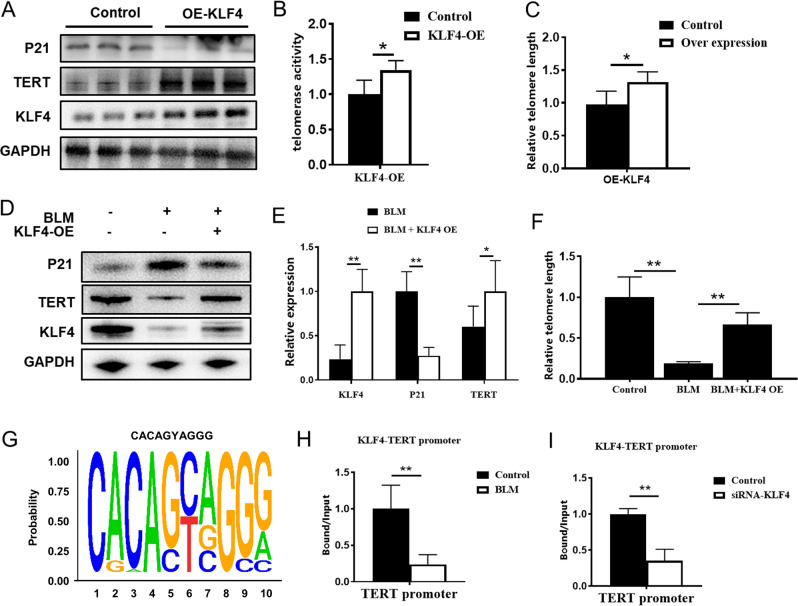
Overexpression of KLF4 Attenuated senescence in AECs and KLF4 regulate TERT. Plko.1 plasmid was used to construct KLF4 overexpression vectors. **A** Western blotting was performed to evaluate the expression of P21, KLF4 and TERT expression in overexpression group and control group in BEAS-2B cells. **B** Telomerase activity was detected by Telomerase Repeated Amplification Protocol (TRAP). **C** Relative telomere lengths were tested in overexpression group and control group in MLE-12 cells. **D** Western blotting was performed to evaluate the expression of P21, KLF4 and TERT expression in overexpression group and control group in MLE-12 cells. **E** Quantification of the protein level of P21, KLF4 and TERT in KLF4 overexpression group and control group in MLE-12 cells. **F** Relative telomere lengths were tested in MLE-12 cells. **G** Chromatin immunoprecipitation sequence (ChIP-seq) results showed that KLF4 can bind to TERT promoter motif. **H**, **I** ChIP-QPCR results indicated that the transcription of KLF4-bound TERT promoter was reduced in bleomycin-induced group and si-RNA group compared with control group. *P < 0.05, **P < 0.01, ***P < 0.001 by *t*-test.

In order to further explore the role of KLF4 in TERT regulation, we used plko.1 plasmid to construct KLF4 overexpression vectors. TRAPEZE XL Telomerase Detection Kit (Millipore, S7707) was used to test the telomere activity. It was found that the expression of TERT and telomerase activity were up-regulated in the KLF4 overexpression group compared with the control group (Fig. 5A–C). Besides, after overexpression of KLF4, the experimental results showed that the expression of senescence markers of bleomycin-induced MLE-12 cells were increased and the expression of TERT was decreased in KLF4 group (Fig. 5A). Our further results showed that in BEAS-2B cells, bleomycin stimulation could result in decreased telomerase activity (*p* = 0.0006) while after KLF4 overexpression, telomerase activity can be protected (*p* = 0.027) (Supplementary Fig. S1). The results indicated that KLF4 had protective effects on TERT expression and telomerase activity.

To further investigate the mechanism of TERT regulation, we established Chromatin immunoprecipitation (ChIP) sequence and ChIP-QPCR in vitro. ChIP-Sequence results showed that KLF4 could bind to TERT promoter motif while it could bind to RTEL and TIN2, which were also telomere-related genes, suggesting that KLF4 may regulate TERT expression through transcription (Fig. 5G). ChIP-QPCR results indicated that the transcription of KLF4-bound TERT promoter was reduced in bleomycin-induced and KLF4 downregulated group, suggesting that the down-regulation of KLF4 in bleomycin treatment would down-regulate the transcription level of TERT, thereby reducing the protein expression of TERT (Fig. 5H, I). These results demonstrate that KLF4 play an important role in TERT and telomere length regulation, probably by modulating the TERT promoter.

## Discussion

In this study, we found that the expression of KLF4 was decreased in AECs in IPF patients and bleomycin-induced pulmonary fibrosis mouse models. Our results showed that KLF4 could protect TERT expression in AECs by binding with the promoter of TERT, and may become a potential target for the treatment of pulmonary fibrosis.

Aging is the main risk factor for developing chronic lung diseases, such as chronic obstructive pulmonary disease, lung cancer, and IPF [27–29]. The pathological manifestations of the lung tissues in IPF are the senescence of AECs and the proliferation of fibroblasts [30]. The occurrence of IPF is related to the long-term repeated damage and abnormal repair of AECs in the early stage of the disease [30]. The premature senescence of AECs is the main cause of epithelial dysfunction [30, 31]. About 97% IPF patients had shortened telomere length [13, 32, 33]. Our previous research found that IPF patients with telomerase gene mutations have significantly shorter telomere length in peripheral blood than those without mutations [11]. Cox proportional hazard model analysis showed that telomere length shortening in peripheral blood nucleated cells was an independent risk factor for survival in patients with IPF, this conclusion was consistent with the findings of a recent study published in the Lancet [34, 35]. A recent study found that alveolar progenitor senescence is sufficient to recapitulate the regenerative defects, inflammatory responses, and susceptibility to injury that are characteristic of telomere-mediated lung disease. They suggest alveolar stem cell failure is a driver of telomere-mediated lung disease and that efforts to reverse it may be clinically beneficial [36]. These studies suggested that telomere shortening-induced cell aging plays an important role in development and prognosis of IPF, and is an important molecular basis for the occurrence of IPF.

TERT expression in epithelial cells was reported to be able to resist bleomycin-induced pulmonary fibrosis [37]. Deficiency of TERT affected the proliferation of epithelial cells, and could result in impaired epithelial regeneration and contribute to enhanced pulmonary fibrosis [37]. Our findings were consistent with previous studies. We found that in bleomycin-induced lung fibrosis models, pulmonary fibrosis were more severe while TERT expression was downregulated. Intervening in the process of cellular aging in AECs could effectively reduce the degree of lung fibrosis in bleomycin-induced fibrosis mouse models, or it may become a reliable therapeutic method to resist aging. In a recent study, the enhanced susceptibility of SPC-*Tert* KO mice to BLM-induced lung injury and fibrosis was not correlated with telomere shortening in AEC II during the 3-week fibrotic period, and telomere shortening was not observed in fibrosis lungs of bleomycin-induced mice, suggested that the precise molecular mechanisms by which TERT protects bleomycin-induced AEC II in a telomere-independent manner requires further elucidation [38]. Consistent with these studies, the current study confirmed the senescence in AECs in IPF lungs, and also proved that the senescence of AECs plays an important role in the progression of IPF [39]. Anti-cellular senescence in the treatment of IPF may lead to better clinical treatment effects.

Here, our results show that the decreased expression of KLF4 in surgical lung biopsy IPF samples than those in normal controls and was decreased in senescent AECs. Our study found that activation of KLF4 in AECs has a positive effect on the maintenance of TERT and telomere length, which may be a potential target for the treatment of pulmonary fibrosis.

KLF4 plays an important role in cellular differentiation and proliferation during normal development and in various diseases. KLF4 belongs to the family of SP/KLF factors which are characterized by three zinc finger motifs within their carboxyl terminal sequences and expression of KLF4 is enriched in intestine, cornea, skin and endothelial cells [15]. As to lung, KLF4 is discovered to be the most significantly altered lung gene at birth and protein product was expressed in fibroblast and airway epithelial cells of perinatal lung tissues of mice [40]. KLF4 is also expressed in pulmonary arterial and venous endothelial cells in mice [41]. The expression of KLF4 was also observed in normal FVB mouse lung tissue and showed decreased level in bleomycin-induced pulmonary fibrosis model. Recently, Chen et al. showed that KLF4 is involved in the regulation of renal physiological functions and the progression of fibrosis [42].

Simultaneous activation of KLF4, OCT3/4, SOX2, and C-MYC can induce reprogramming of different types of differentiated cells into pluripotent stem cells (iPS) [43]. In stem cells, β-catenin binds to KLF4 to form a complex and binds to the promoter region of TERT gene to regulate TERT gene expression and promote telomere lengthening [23]. The reduction of KLF4 in human embryonic stem cells reduces the expression of TERT, which replaces the function of KLF4 and maintains the self-renewal of embryonic stem cells. Therefore, KLF4 maintains TERT expression by directly activating the transcription of TERT in embryonic stem cells, which also explains why KLF4 can promote the induction of differentiated cells into pluripotent stem cells [19]. Our results also showed that KLF4 could protect telomerase activity meanwhile. Elongation of telomeres requires telomerase access to telomeric DNA. According the literature, telomerase activity remains active mainly in stem cells, such as male germ cells, activated lymphocytes, and certain types of stem cells [44]. Crucial regulation of telomerase activity occurs through the control of TERT transcription, which dictates TERT levels [45]. Telomerase activity is regulated by telomerase Cajal body protein 1 (TCAB1). It has been reported that binding of TCAB1 promotes a catalytically active state of telomerase by enhancing RNA folding and encouraging proper association of TERT and telomere RNA [46]. As TPP1–POT1 binds to single-strand DNA through POT1, the initial prediction was that telomerase and TPP1–POT1 would compete for telomere binding [47]. In some stem cells, TERT promoter mutations activate TERT transcription, resulting in increased mRNA levels, promoted telomerase activity and increased telomere length [48].

Activation of KLF4 has been reported to have anti-fibrotic effects in different types of cells in lung. KLF4 in epithelial cell can inhibit TGF-β1-induced EMT by down-regulating SNAI2 [21]. KLF4 in macrophages can alleviate TNF-α -mediated injury by inhibiting EMT [49]. In lung-resident mesenchymal stem cells (LR-MSCs), KLF4 transcription level was downregulated after LR-MSC stimulated by TGF-β1 while LR-MSC could promote lung fibrosis by differentiating into myofibroblasts [50]. Our data further confirmed that KLF4 is decreased in AECs of IPF lungs and bleomycin-induced pulmonary fibrosis. Our data provide further evidence to support KLF4 as a promising anti-fibrotic transcriptional factor. Further studies are needed to explore the role of KLF4 in IPF lung fibrosis.

In conclusion, the present study demonstrates that the transcription factor KLF4 attenuates bleomycin-induced lung fibrosis by protecting TERT expression and telomere length. KLF4 could be a promising potential target for further understanding the mechanism and developing novel strategy for the treatment of lung fibrosis.

## Methods

### Ethics statement

12 patients with IPF were included in this study and 12 cancer adjacent normal lung tissues were included as control. IPF diagnoses was accomplished by pulmonologists according to 2018 American Thoracic Society guideline [1]. These IPF patients had no history of cancer or other lung diseases. The lung tissues of IPF and control groups were obtained from Nanjing Drum Hospital and Wuxi People’s Hospital. All animal and human experiments protocols were approved by the Ethics Committee of Nanjing Drum Tower Hospital and informed written consents were obtained from all participants involved in this study.

### Bleomycin-induced fibrosis model

All experimental animal procedures were conducted in accordance with humane animal care standards with approval from the Nanjing Drum Tower Hospital Ethics Committee. Male C57/B6 mice with an average weight of ~25–30 g and aged from 6 months weeks were intratracheally instilled with saline or 5 mg/kg dissolved in a total of 50 μl sterile saline. Control group was similarly treated with 50 μl of sterile saline. Mice were killed on the day 14 and lung tissues were collected for further analysis. Lungs were fixed in formalin and embedded in paraffin, sectioned and stained with hematoxylin and eosin (H&E) and Masson staining, or immunohistochemistry with antibodies. All experiments were performed in accordance with relevant guidelines and regulations.

### Construction of specific overexpression of KLF4 mice model

The adeno-associated viral (AAV)−6 vectors mediated KLF4 over-expression with SP-C promoter was constructed by Genechem, Shanghai. The mice were randomized into groups and there was five mice in every group. It was intratracheally instilled to 8 weeks old mice. Wild-type littermates were used as controls. No difference in weight or survival rate was observed between overexpression group and wild type group. Mice were housed in a constant temperature and humidity-controlled specific pathogen-free facility, with standard chow and water ad libitum.

### Cell culture

MLE-12 cells and BEAS-2B cells were obtained from the American Type Culture Collection (Manassas, VA). Cells were cultured in the same medium (DMEM/F12) with 10% FBS to avoid any growth variability. Cells passaging were performed when they reached 80% confluence and the medium is changed every two days. All cell lines were authenticated by STR profiling and there was no mycoplasma contamination.

### Small-interfering RNA (siRNA)-mediated gene knockdown

The siRNA targeting mouse KLF4 mRNA (NM_004235) was synthesized with the sense sequence 5’-AGACGCUUCCAAGUUAUAU-3’. SiRNA for KLF4 and scrambled siRNA were purchased from Ruibo (Shanghai, China). The double-strand RNAs (100 nM) were transfected into cells with lipofectamine 2000 (Invitrogen). The control siRNA was used at the same dose.

### Western blot analysis

Western blots were performed to verify the expression of proteins in control and IPF group. Total protein extracts (20 μg) were separated on 10% SDS-PAGE and transferred to nitrocellulose membranes. The blots were probed with anti-KLF4 (Abcam, Ab214666), anti-TERT (Abcam, Ab32020) and anti-GAPDH (Abcam, Ab8245) antibodies. The signal was detected by enhanced automatic chemiluminescence camera (Tanon 5200).

### Quantitative reverse transcription-polymerasechain reaction (qRT-PCR)

Total RNA was extracted from human or mouse lung tissues or cultured cells with Trizol reagent (Vazyme, Nanjing, China). The HiScript 1^st^ Strand cDNA Synthesis Kit (Vazyme) was used for reverse transcription PCR (RT-PCR). Comparative quantitative PCR (Q-PCR) was performed by using the SYBR Green Q-PCR Kit (Roche, Germany) with GAPDH used as an internal control. Primers are listed in Supplementary Table S2. The Ct values were analyzed using the ΔΔCt method.

### Senescence-associated β-galactosidase assay

The Senescence β-Galactosidase Staining Kit was purchased from Beyotime Biotechnology (Shanghai, China). SA-β-Gal staining was performed according to the manufacturer’s protocol. Briefly, cell samples or frozen lung tissue sections were fixed with 4% formaldehyde for 10 min at room temperature. The slides were rinsed with PBS, followed with the incubation of freshly prepared SA-β-Gal staining solution overnight. Then, the cells or tissue sections were washed with PBS at room temperature. The images were further captured by using a microscope equipped with a digital camera (OLYMPUS CKX41).

### Immunohistochemistry and immunofluorescence

Lung tissues were fixed with 4% paraformaldehyde and embedded with paraffin. Cells were cultured on cover slips and subjected to staining. Sections were incubated with the appropriate primary antibodies at 37 °C for 1 h and 4 °C overnight. The secondary antibody was incubated for 1 h at 37 °C. Sections were viewed with light microscopy. Negative controls were performed by omitting the primary antibody. As to phalloidin staining, F-actin was stained with FITC-conjugated phalloidin at a 1:1000 dilution in 2% BSA for at least 30 min. Hoechst was used to counterstain the nuclei. Images were acquired with confocal laser scanning microscope. As to statistical analysis of double-staining positive cells, the confocal photos were taken under FV3000 confocal microscopy (OLYMPUS) randomly and at least 5 visual fields were included in each section for further calculation.

### Chromatin immunoprecipitation (ChIP) assay

Cells for ChIP were cultured in 10 × 10 cm dishes. The ChIP assay was performed by following the instructions of the ChIP Kit (CST #S58676). MLE12 cells were cross-linked with 1% formaldehyde, followed by 0.1 M glycine to stop the reaction. Then, the chromatin was sheared into fragments of 500–1000 bp in length by ultrasound and the DNA-protein complex of chromatin fragments was precipitated by anti-KLF4 or anti-IgG antibody. The DNA was then eluted and extracted with phenol-chloroform and amplified by Q-PCR or for CHIP-seq. TERT promoter-specific primers were used to amplify the KLF4 binding regions. The primers were as follows: TERT promoter sense, 5’-TCATCTGAACCTCCTCTC-3’, TERT promoter antisense, 5’-GTTGCTTGATTTGAA TTTGAG-3’.

### Telomere length measurement

The relative repeat copy number of telomere (T) and single gene (36B4a) copy number (S) were determined by real-time polymerase chain reaction (PCR) with power SYBR green PCR master mix (Applied Biosystems, Carlsbad, CA, USA) using a Step One Plus TM Real-Time PCR System (Applied Biosystems). The primer sequences were as follows: Telomere—Forward, ACACTAAGGTTTGGGTTTGGGTTTGGGTTTGGGTTAGTGT; Reverse, TGTTAGGTATCCCTATCCCTATCCCTATCCCTATCCCTAACA; 36B4—Forward, CAGCAAGTGGGAAGGTGTAATCC; Reverse, CCCATTCTATCATCAACGGGTACAA10. Each sample was analyzed in triplicate. To perform standard curves, one reference DNA was serially diluted in two-fold steps with deionized water, to create eight concentrations of DNA ranging from 1.875 to 240 ng/μL. The R2 of the standard curves was >98%, and amplification efficiency of each primer pair was >98%. The relative TL was calculated as the T/S ratio, reflecting the average telomere repeat copy number of each DNA sample calculated relative to the reference DNA.

### Telomerase activity measurement

The TRAPEZE Kit (Millipore, Cat #S7707) was used to detect the telomerase activity by following the instructions. The cell pellets were suspended into CHAPS Lysis Buffer and then incubated the suspension on ice for 30 min. Then spin the sample at 12,000 × *g* for 20 min at 4 °C and transfer supernatant to determine the protein concentration. Prepare “Master Mix” by mixing the reagents including TRAPEZE reaction mix, taq polymerase, dH_2_O and cell extract. After incubating at 30 °C for 30 min, 4-step PCR was performed at 94 °C/30 s, 59 °C/30 s, 72 °C/1 min for 35 cycles followed by a 72 °C/3 min extension step and then at 55 °C/25 min, concluding with a 4 °C incubation. The PCR reactions were determined by measuring the fluorescence in a spectrofluorometer. The TSR8 standard curve was generated and the TPG value for each sample was obtained.

### Statistical analysis

All assays were performed in at least triplicates and repeated a minimum of 3 times. Results are expressed as mean ± standard deviation (SD). Significant differences between means compared to the control were determined using Student’s *t* test and *p* < 0.05 was considered significant.

## Supplementary information

Supplementary

Original Data File

Cover Art

Cover Art

## Data Availability

The data that support the findings of this study are available from the corresponding author upon reasonable request.
