# Supplementary material for: KLF4 regulates TERT expression in alveolar epithelial cells in pulmonary fibrosis
Source: Cell Death Dis. 2022 May 4;13(5):435. doi: 10.1038/s41419-022-04886-7 (PMC9068714; doi:10.1038/s41419-022-04886-7)
Supplement: Supplementary file 1 — Supplementary [file 41419_2022_4886_MOESM1_ESM.docx]

**Table S1. The demographic data of IPF patients and control samples**

|  | IPF (n=12) | Control (n=12) | p |
| --- | --- | --- | --- |
| Female | 1 (8.33%) | 2 (16.67%) | 1.000 |
| Average age | 57.5±3.99 | 56.17±5.10 | 0.457 |
| Smoking history | 8 (66.67%) | 7 (58.33%) | 0.673 |

**Table S2. qPCR primers and ChIP-qPCR sequence**

|  | Primer | Sequence |
| --- | --- | --- |
| M-GAPDH | Front | AGGTCGGTGTGAACGGATTTG |
|  | Reverse | TGTAGACCATGTAGTTGAGGTCA |
| M-KLF4 | Front | GGCGAGTCTGACATGGCTG |
|  | Reverse | GCTGGACGCAGTGTCTTCTC |
| M-TERT | Front | TCTACCGCACTTTGGTTGCC |
|  | Reverse | CAGCACGTTTCTCTCGTTGC |
| M-P21 | Front | CCTGGTGATGTCCGACCTG |
|  | Reverse | CCATGAGCGCATCGCAATC |
| M-P16 | Front | CGAACTCGAGGAGAGCCATC |
|  | Reverse | TACGTGAACGTTGCCCATCA |
| H-GAPDH | Front | TCAACAGCAACTCCCACTCTTCCA |
|  | Reverse | ACCCTGTTGCTGTAGCCGTATTCA |
| H-KLF4 | Front | CCCACATGAAGCGACTTCCC |
|  | Reverse | CAGGTCCAGGAGATCGTTGAA |
| H-TERT | Front | TCACGGAGACCACGTTTCAAA |
|  | Reverse | TTCAAGTGCTGTCTGATTCCAAT |
| H-P21 | Front | TGTCCGTCAGAACCCATGC |
|  | Reverse | AAAGTCGAAGTTCCATCGCTC |
| H-p16 | Front | CTTCCTCGGGTGCCGATAC |
|  | Reverse | ACCCCTTCATTGCTACTCGAT |
| TERT-promoter | Front | AGTCTAGACCACCTGGGGATTC |
|  | Reverse | GTGCTTAGAAAACAGCCAGGAC |

**Figure S1: Telomerase activity test in BEAS-2B cells.**

TRAPEZE XL Telomerase Detection Kit (Millipore, S7707) to test the telomere activity. Results showed that bleomycin stimulation could result in decreased telomerase activity (p=0.0006) while after KLF4 overexpression, telomerase activity can be protected (p=0.027). *P<0.05, **P<0.01, ***P < 0.001 by t-test.
